# PSCA rs2294008 Polymorphism with Increased Risk of Cancer

**DOI:** 10.1371/journal.pone.0136269

**Published:** 2015-08-26

**Authors:** Peiliang Geng, Jianjun Li, Ning Wang, Juanjuan Ou, Ganfeng Xie, Chen Liu, Xiaoxin Zhao, Lisha Xiang, Yunmei Liao, Houjie Liang

**Affiliations:** Department of Oncology and Southwest Cancer Center, Southwest Hospital, Third Military Medical University, Chongqing, 400038, China; National Cancer Center, JAPAN

## Abstract

**Background:**

Published data on the association between *PSCA* rs2294008 polymorphism and cancer risk have implicated inconclusive results. To determine the relationship and to precisely assess the effect size estimate of the association, we performed a meta-analysis.

**Methods:**

We searched published literature in Embase and PubMed databases using the search terms “*PSCA*”, “prostate stem cell antigen”, “variants”, “polymorphism”, “polymorphisms”, and “cancer”. A total of 21 eligible articles were retrieved, with 27, 197 cancer cases and 48, 237 controls.

**Results:**

On the whole, we found the association between *PSCA* rs2294008 polymorphism and cancer risk was statistically significant: TT vs CC: OR = 1.18, 95% CI, 1.10 to 1.27; TT + CT vs CC: OR = 1.08, 95% CI, 1.05 to 1.10; TT vs CT + CC: OR = 1.14, 95% CI, 1.07 to 1.21; T vs C: OR = 1.10, 95% CI, 1.06 to 1.14; CT vs CC: OR = 1.10, 95% CI, 1.06 to 1.13. Stratified analyses in cancer type and ethnicity showed similar results.

**Conclusions:**

Based on the statistical evidence, we can draw a conclusion that the rs2294008 polymorphism of PSCA gene is likely to play a role in cancer carcinogenesis, especially in gastric cancer and bladder cancer.

## Introduction

Genome-wide association studies (GWAS) concerning aetiology of cancer have established more than 150 regions associated with various specific cancers. The discoveries successfully expand the current understanding of carcinogenesis mechanisms [1]. Given the functional significance of genetic polymorphisms in cancer initiation and progression, it is of great importance to further explore the underlying pathophysiology of cancer at the gene level. Genetic variations, such as the alterations in sequence and aberrant organizations of the cellular genome ranging from single-nucleotide substitutions to gross chromosome, lead to cancer formation by biologically regulating a handful of molecular activities.

Prostate stem cell antigen (*PSCA*), located on chromosome 8q24.2, is a 123-amino-acid cell membrane glycoprotein which belongs to the LY-6/Thy-1 family of cell surface antigens. *PSCA* was reported as a cell surface marker to over-express in prostate cancer cell lines when compared to the normal tissues [2]. High expression of *PSCA* is significantly associated with adverse prognostic features including Gleason score, seminal vesicle invasion and capsular involvement [3], as well as cancer severity and metastasis [4]. In addition, *PSCA* is up-regulated in several solid tumors (pancreas, bladder, renal cell carcinoma and ovarian mucinous) [2,5], and down-regulated in esophagus cancer, gastric cancer and gallbladder carcinoma [6–8]. However, there is no conclusive evidence for the role of *PSCA* expression in cancer carcinogenesis except a proposal that the expression of *PSCA* differs depending on cellular context [5].

Two recent GWAS based on the subjects with different ethnic origins showed rs2294008 polymorphism in *PSCA* is a significant risk factor for increased gastric cancer susceptibility in Caucasian population [9], while deceases gastric cancer risk in Asian population [10]. rs2294008 polymorphism in the first exon of *PSCA* gene may have notable influence on the variations in transcriptional activity of an upstream fragment of *PSCA* [8]. To date, although accumulating data have documented the association between *PSCA* rs2294008 polymorphism and cancer risk, the evidence regarding the role of the polymorphism as a genetic marker for cancer risk remains inconclusive [9–14]. Most of the studies focused on a single type of cancer with a relatively small sample size. As a result, the effects of *PSCA* rs2294008 polymorphism on cancer risk may be underestimated and less reliable. In this work, therefore, with an aim to determine the relationship between *PSCA* rs2294008 polymorphism and cancer risk and to precisely assess the effect size estimate of the association, we performed a meta-analysis using all available published data.

## Materials and Methods

### Search strategy

Studies examining the association between *PSCA* rs2294008 polymorphism and cancer risk were comprehensively searched in Embase and PubMed using the following subjects terms: “*PSCA*”, “prostate stem cell antigen”, “variants”, “polymorphism”, “polymorphisms”, and “cancer” by two independent investigators. No limitations of publication language or a minimum number of subjects were defined for this search. Additional published data were identified by reviewing the bibliographical references listed in each retrieved article.

### Inclusion and exclusion criteria

In order to minimize heterogeneity and facilitate an appropriate interpretation of the findings, we selected the studies eligible for this meta-analysis based on the following criteria: (a) assessed the association between *PSCA* rs2294008 polymorphism and cancer risk using a case-control design; (b) provided available frequency for each genotype (CC, CT, TT) in both cases and controls to calculate odds ratios (ORs) and corresponding 95% confidence intervals (CIs). In a case that a same case series was subsequently used in a new article, we considered the largest one. The studies were excluded if they were: reviews, editorials, comments or animal studies.

### Data extraction

The data extraction was completed by the two independent investigators responsible for the literature search. The information collected form each publication was as follows: first author’s name, year of publication, study country, ethnic origin of the included subjects (Caucasian or Asian), methods conducted for genotyping, source of controls (hospital- or population-based), type of cancer, total numbers of cases and controls, and frequency of *PSCA* rs2294008 genotypes between cases and control subjects. A consensus on the extracted items was reached by discussion between the two investigators.

### Statistical analysis

In order to evaluate the association between *PSCA* rs2294008 polymorphism and cancer risk, the ORs and corresponding 95% CIs were summarized for each study in TT vs CC, TT + CT vs CC, TT vs CT + CC, T vs C and CT vs CC models. Subgroup analysis was performed according to ethnicity and cancer type (gastric cancer, bladder cancer and others when concerned by less than three studies).

Heterogeneity is the degree of variations among outcomes between different studies included in a same meta-analysis. Chi-square based Q-test was adopted to detect the heterogeneity across studies in this meta-analysis. *P* <0.10 was considered statistically significant. In this case, the pooled ORs were calculated by the random effects model (Der Simonian and Laird) [15]; otherwise, the fixed effects model (Mantel-Haenszel method) was used [16].

Sensitivity analysis was performed to identify the study that influenced homogeneity of the included studies when significant heterogeneity was indicated. Hardy-Weinberg equilibrium (HWE) in the control group was checked by chi-square test. Publication bias was examined by two commonly used analytic tools: funnel plot and Egger’s linear regression test [17].

The statistical analyses for the present study were done by using Stata software (version 12.0; StataCorp LP, College Station, TX, USA). All tests were two-sided and significance level was maintained at *P* <0.10.

## Results

### Study selection

**Fig 1:**
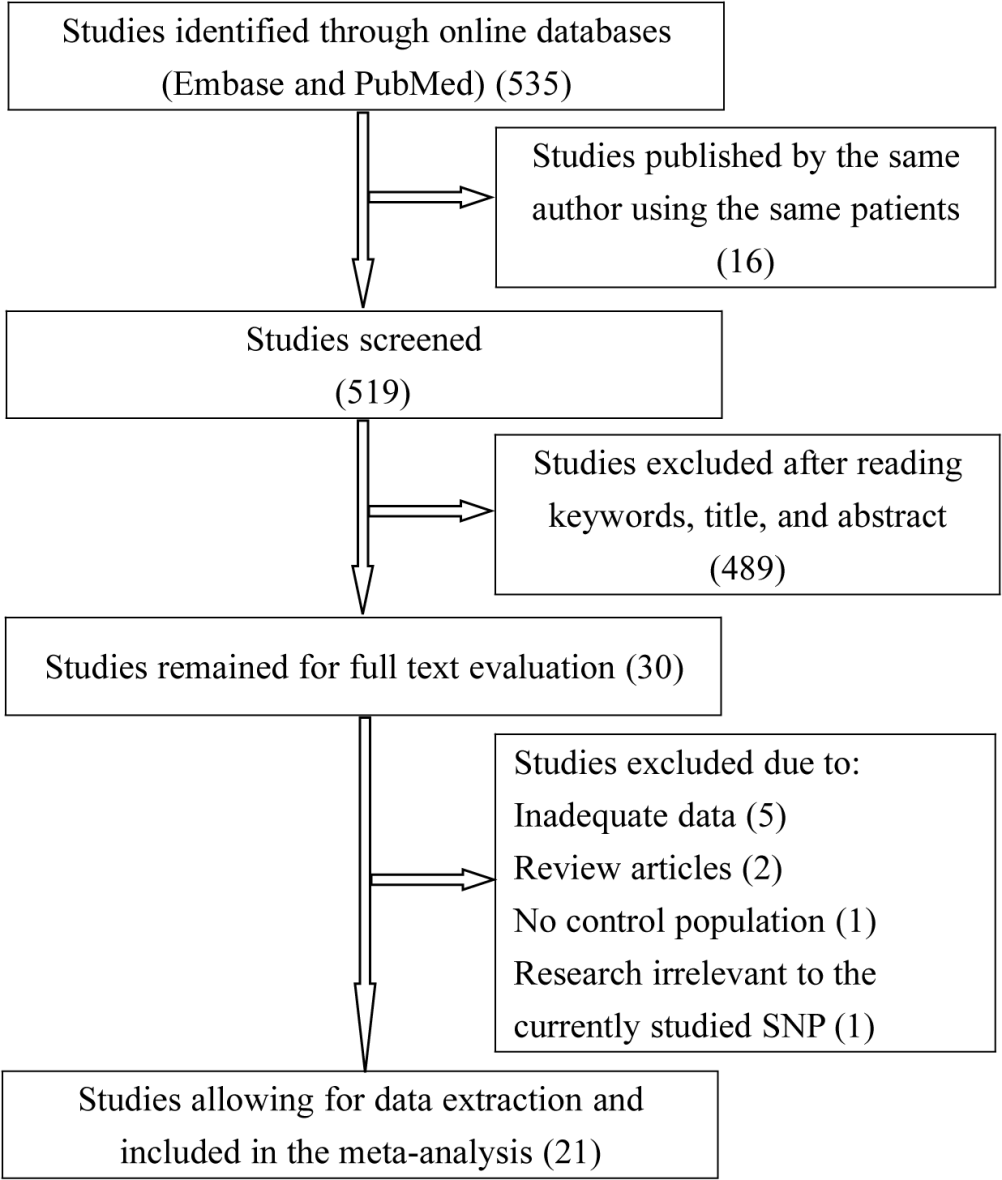
Flow diagram of the study selection process.

We obtained 535 articles matching the subjects term used in the search strategy. After reviewing their titles and abstracts, we selected 30 articles in full text for eligibility evaluation. Among these, 9 were finally deleted for various reasons including inadequate data [18–22], review articles [23,24], with no control population [25] and research irrelevant to the currently studied polymorphism [26]. Therefore, 27, 197 cancer cases and 48, 237 controls from 21 articles with 24 studies were included in this meta-analysis [4,8–14,27–39] (Fig 1).

### Characteristics of included studies

The basic characteristics of all included studies are listed in Table 1. A total of twenty four studies were eligible for this meta-analysis, among which eight were for Caucasian subjects and sixteen for Asian subjects. There was wide differences in the literature as to the number of participants in each study, varying from 77 to 5, 303 in case group and from 200 to 16, 567 in control population. Only two studies deviated from the p values of HWE [10,33].

**Table 1:** Main characteristics of all studies included in the meta-analysis. PCR-RFLP: polymerase chain reaction-restriction fragment length polymorphism; PCR-LDR: PCR-ligation detection reaction; DHPLC: denaturing high performance liquid chromatography; AS-PCR: allele-specific PCR; SNP: single nucleotide polymorphism, PB: population-based studies; HB: hospital-based studies.

Study	Year	Area	Ethnicity	Genotyping method	Cancer type	Control source	No. of cases	No. of controls
Sakamoto et al.	2008	Japan	Asian	TaqMan, SNP array	Gastric cancer	PB	1524	1396
Sakamoto et al.	2008	Korea	Asian	TaqMan, SNP array	Gastric cancer	PB	871	390
Matsuo et al.	2009	Japan	Asian	TaqMan	Gastric cancer	HB	708	708
Wu et al.	2010	China	Asian	PCR-RFLP	Gastric cancer	HB	1710	995
Ou et al.	2010	China	Asian	PCR-LDR	Gastric cancer	HB	196	246
Lu et al.	2011	China	Asian	PCR-RFLP	Gastric cancer	PB	1023	1069
Song et al.	2011	Korea	Asian	PCR-RFLP	Gastric cancer	HB	3245	1700
Zeng et al.	2011	China	Asian	PCR-RFLP	Gastric cancer	HB	460	549
Lochhead et al.	2011	Polish	Caucasian	TaqMan	Gastric cancer	PB	292	382
Lochhead et al.	2011	USA	Caucasian	TaqMan	Gastric cancer	PB	308	208
Sala et al.	2011	Spain	Caucasian	SNP array	Gastric cancer	PB	409	1515
Zhao et al.	2012	China	Asian	DHPLC	Gastric cancer	PB	185	200
Li et al.	2012	China	Asian	iPLEX	Gastric cancer	HB	300	300
Tanikawa et al.	2012	Japan	Asian	SNP array	Gastric cancer	HB	2300	16567
Rizzato et al.	2013	Venezuela	Caucasian	AS-PCR	Gastric cancer	HB	178	1057
Wu et al.	2009	USA	Caucasian	SNP array	Bladder cancer	PB	5038	9363
Wang et al.	2010	China	Asian	PCR-RFLP	Bladder cancer	HB	581	580
Fu et al.	2012	Spain	Caucasian	SNP array	Bladder cancer	PB	5393	7324
Ma et al.	2013	China	Asian	iPLEX	Bladder cancer	PB	175	962
Wang et al.	2013	China	Asian	TaqMan	Bladder cancer	PB	1210	1008
Lochhead et al.	2011	USA	Caucasian	TaqMan	Esophageal cancer	PB	158	208
Rai er al.	2013	India	Asian	TaqMan	Gallbladder carcinoma	PB	405	247
Kim et al.	2012	Korea	Asian	AS-PCR	Breast cancer	HB	451	459
Smith et al.	2012	UK	Caucasian	TaqMan	Colorectal cancer	PB	77	804

### Quantitative synthesis

**Fig 2:**
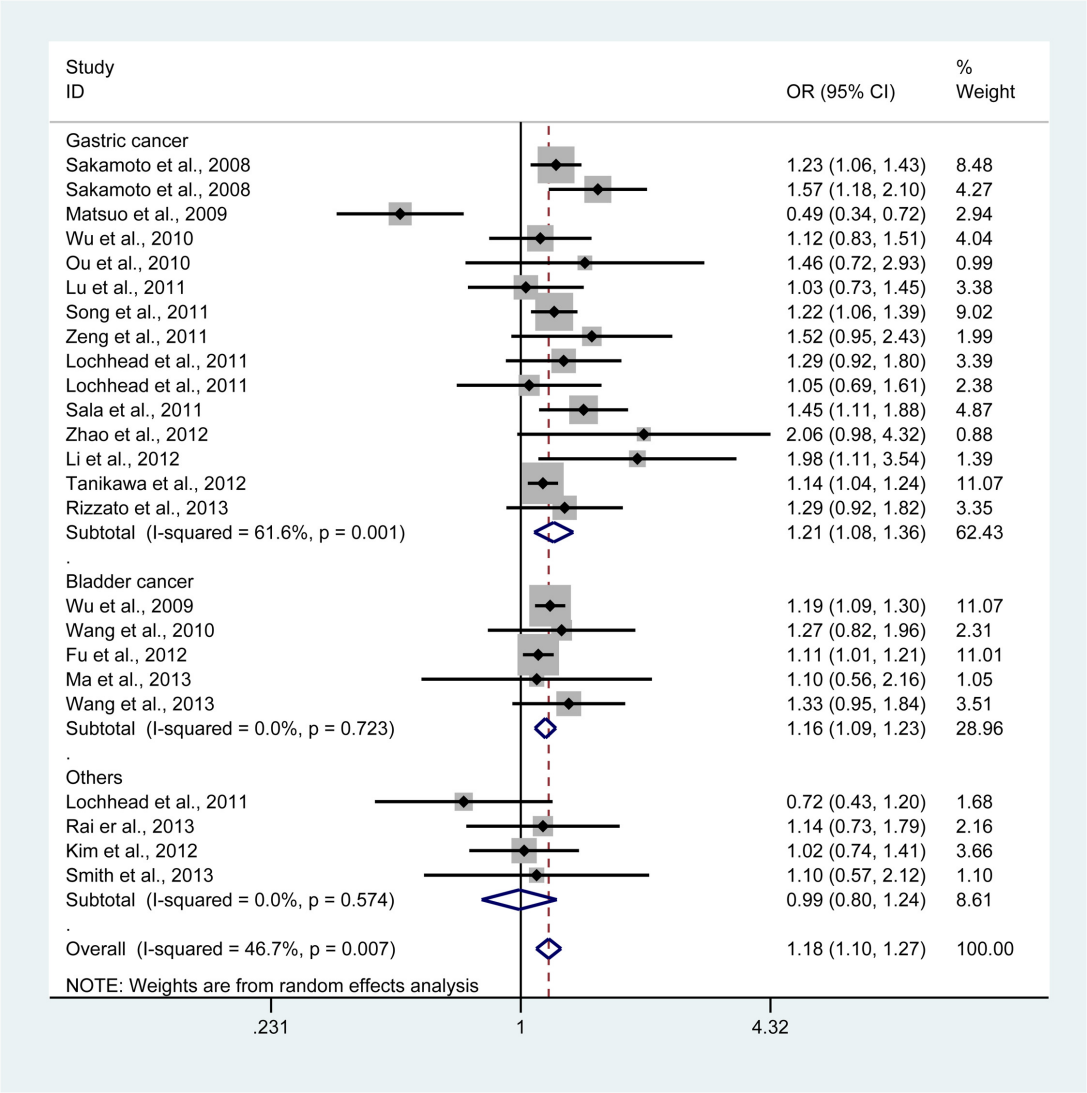
Forest plot of cancer susceptibility associated with *PSCA* rs2294008 polymorphism under TT vs CC stratified according to cancer type. For each study, the estimates of OR and its 95% CI were plotted with a box and a horizontal line. The symbol filled diamond indicates pooled OR and its 95% CI. Random effects meta-analysis shows a significant association.

**Fig 3:**
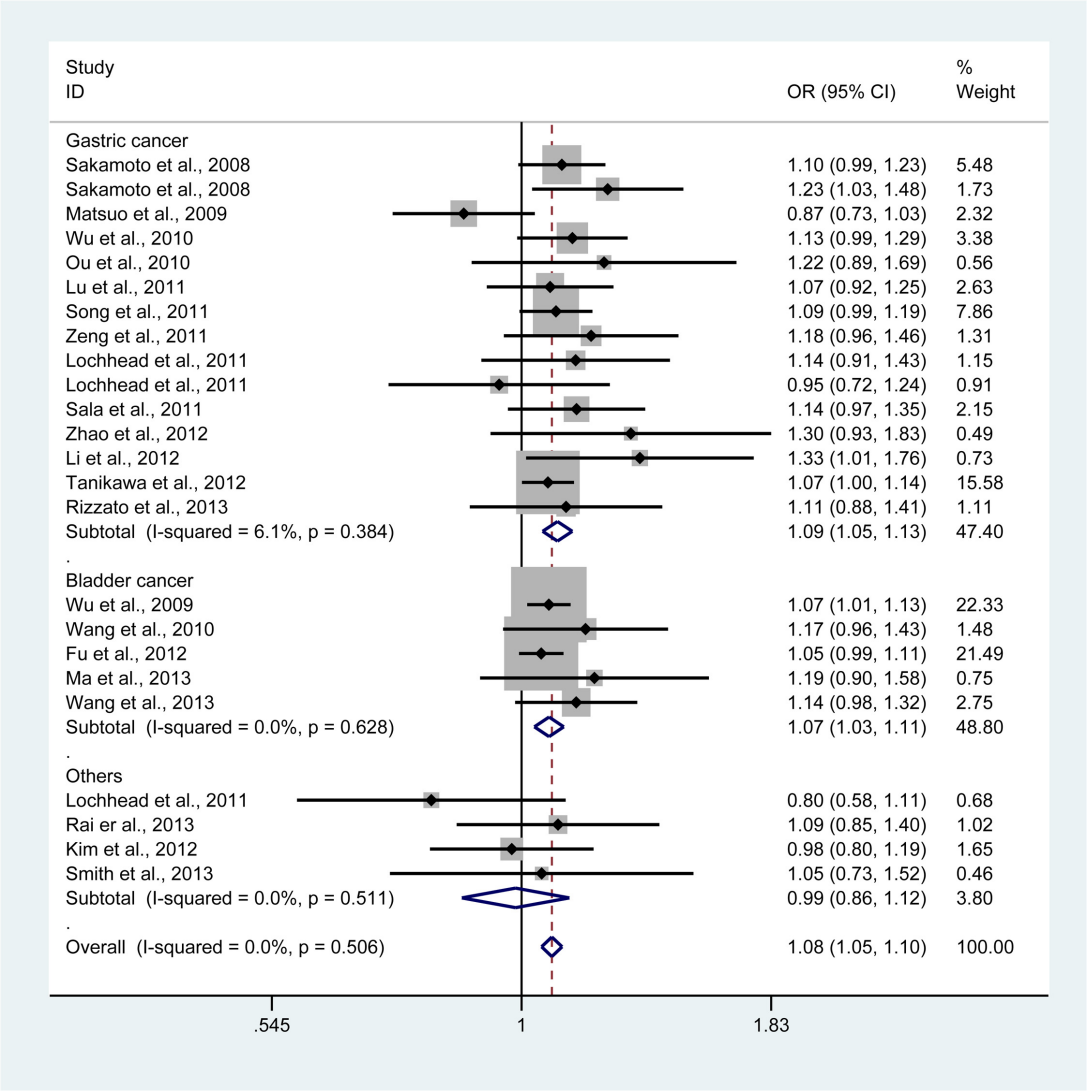
Forest plot of cancer susceptibility associated with *PSCA* rs2294008 polymorphism under TT + CT vs CC stratified according to cancer type. For each study, the estimates of OR and its 95% CI were plotted with a box and a horizontal line. The symbol filled diamond indicates pooled OR and its 95% CI. Fixed effects meta-analysis shows a significant association.

By pooling all eligible studies into one large dataset, we found the association between *PSCA* rs2294008 polymorphism and cancer risk was statistically significant. The association appeared more pronounced in TT vs CC and TT vs CT + CC: OR = 1.18, 95% CI, 1.10 to 1.27 (Fig 2); OR = 1.14, 95% CI, 1.07 to 1.21, respectively. We also noted a moderate increase using TT + CT vs CC (OR = 1.08, 95% CI, 1.05 to 1.10) (Fig 3), T vs C (OR = 1.10, 95% CI, 1.06 to 1.14), and CT vs CC (OR = 1.10, 95% CI, 1.06 to 1.13) (Table 2).

**Table 2:** Results of meta-analysis for PSCA rs2294008 C>T polymorphism and cancer. P, p value of Q test for heterogeneity.

Study group	No. of studies	TT vs CC		TT + CT vs CC		TT vs CT + CC		T vs C		CT vs CC	
		OR (95% CI)	P	OR (95% CI)	P	OR (95% CI)	P	OR (95% CI)	P	OR (95% CI)	P
Total	24	1.18 (1.10, 1.27)	0.007	1.08 (1.05, 1.10)	0.506	1.14 (1.07, 1.21)	0.029	1.10 (1.06, 1.14)	0.007	1.10 (1.06, 1.13)	0.497
Cancer type											
Gastric cancer	15	1.21 (1.08, 1.36)	0.001	1.09 (1.05, 1.13)	0.384	1.17 (1.06, 1.29)	0.004	1.12 (1.07, 1.18)	0.004	1.12 (1.08, 1.18)	0.536
Bladder cancer	5	1.16 (1.09, 1.23)	0.723	1.07 (1.03, 1.11)	0.628	1.10 (1.04, 1.17)	0.699	1.08 (1.05, 1.11)	0.481	1.08 (1.04, 1.13)	0.673
Others	4	0.99 (0.80, 1.24)	0.574	0.99 (0.86, 1.12)	0.511	1.04 (0.85, 1.27)	0.909	1.00 (0.90, 1.11)	0.475	0.97 (0.84, 1.13)	0.351
Ethnicity											
Asian	16	1.18 (1.06, 1.32)	0.003	1.09 (1.06, 1.13)	0.397	1.12 (1.02, 1.23)	0.013	1.11 (1.06, 1.17)	0.006	1.13 (1.08, 1.17)	0.609
Caucasian	8	1.17 (1.08, 1.26)	0.309	1.06 (1.02, 1.10)	0.655	1.13 (1.06, 1.20)	0.367	1.08 (1.03, 1.12)	0.245	1.07 (1.03, 1.11)	0.473

Stratification analyses for different cancer type showed similarly increased risk in gastric cancer and bladder cancer. When stratifying the populations according to ethnicity, both Asian and Caucasian populations indicated a tend to an increase in the risk of cancer (Table 2).

### Heterogeneity and sensitivity analyses

Due to significant heterogeneity across studies (TT vs CC: *P* = 0.007; TT vs CT + CC: *P* = 0.029; T vs C: *P* = 0.007), sensitivity analysis by repeating the meta-analysis while omitting each study, one at a time, was conducted to identify the source. The results showed Matsuo et al. [27] made major contributions to the notable heterogeneity. The exclusion of this study dramatically decreased the heterogeneity (TT vs CC: *P* = 0.440; TT vs CT + CC: *P* = 0.710; T vs C: *P* = 0.245). However, the combined results were not statistically influenced by excluding each study, including the study in disagreement with HWE. These data suggested that our results are stable and robust.

### Publication bias

**Fig 4:**
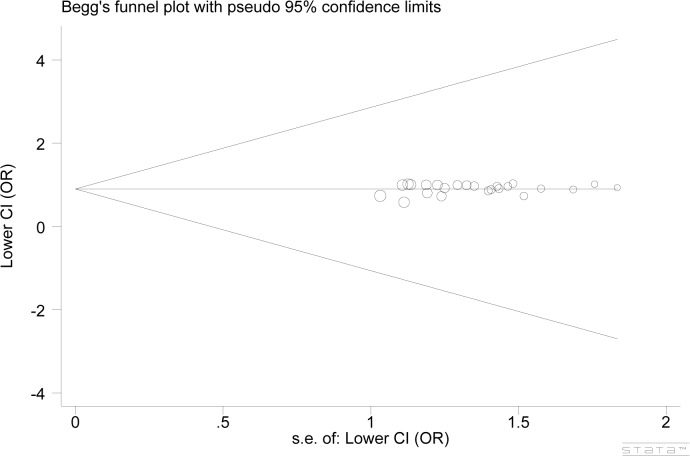
Funnel plots of *PSCA* rs2294008 polymorphism and cancer risk (Begg: *P* = 0.442; Egger: *P* = 0.316; model: TT + CT vs CC). Each point represents an individual study for the indicated association.

Begg’s funnel plot and Egger’s test were used to determine publication bias in this meta-analysis. There was no obvious asymmetry indicated in the plots. Egger’s test also provided supportive evidence for no significant publication bias in the meta-analysis (Begg: *P* = 0.442; Egger: *P* = 0.316; model: TT + CT vs CC) (Fig 4).

## Discussion

*PSCA* as a prostate-specific antigen plays a key role in cell adhesion, proliferation, and survival [40]. The serum level was directly or indirectly associated with cancer progression. *PSCA* over-expression was initially reported in prostate cancer [2], followed by several other cancers, such as pancreatic cancer [41,42]. Investigations into the association between *PSCA* rs2294008 polymorphism and cancer risk have been frequently conducted in either a small or large population to identify the relationship. Currently, there is no consistent evidence supporting this association. Some studies indicated the rs2294008 polymorphism of *PSCA* gene may have a significant role in bladder carcinogenesis and it could serve as a biomarker for genetic susceptibility to this cancer [37,38], while this finding contradicted a following replication study regarding gastric cancer in Japanese population, where a reduced risk of gastric cancer was suggested [10]. A most recent study, however, even demonstrated no genotypic association between *PSCA* rs2294008 polymorphism and the risk of colorectal cancer. In view of the relatively limited sample size, it is indefinite to determine a stable effect size of *PSCA* rs2294008 polymorphism in relation to cancer.

Meta-analysis has been widely accepted as a significant tool to analyze cumulative data from studies in which results with low statistical power were produced due to the limited study subjects [43]. In the present meta-analysis, composed of 27, 197 cancer cases and 48, 237 controls from 21 articles with 24 studies, comprehensively explored the association between *PSCA* rs2294008 polymorphism and cancer risk. The combined results suggested a statistical association with overall cancer risk. In the stratified analyses according to cancer type, elevated risks of gastric cancer and bladder cancer were indicated. Consistent with the findings in the former analyses, stratification analyses in ethnicity also showed *PSCA* rs2294008 polymorphism was associated with an increased risk of cancer in Asian as well as Caucasian populations.

This is the first study addressing cancer risk associated with *PSCA* rs2294008 polymorphism to date, although a number of meta-analyses have investigated the predisposition effects of *PSCA* rs2294008 polymorphism on gastric cancer [6,060 cases and 4,824 controls, 10,717 cases and 9,028 controls, 10,836 cases and 9,235 controls in the study conducted by Wang et al., Zhang et al. (Asian Pac J Cancer Prev), Zhang et al. (Exp Ther Med), respectively] [44–46]. All of these studies showed PSCA rs2294008 polymorphism is a risk factor for the development of gastric cancer, an observation consistent with the current work (55,363 more participants even compared to the largest study) which was expanded via the inclusion of several subsequently published studies. The reference to increased cancer susceptibility implies that variation of rs2294008 polymorphism leads to abnormal expression of PSCA, consequently promoting cancer progression regardless of cancer type or ethnic origin.

It is known that H. pylori infection represents a main and specific infectious cause of human cancer, gastric cancer in particular. Various lines of evidence have demonstrated the important role H. Pylori infection plays in gastric cancer. For example, Levi et al. reported that not only H. pylori infection itself acts as a susceptibility factor, but it may have additive effects on gastric carcinogenesis by combining with other confounding factors [47]. Almost at the same time, Wang et al. provided further evidence that persistent H. pylori infection and the infection-induced chronic inflammation may increase the likelihood of gastric cardia cancer in Chinese [48]. Also, Lunet et al. indicated that H. Pylori has a major impact with the effects varying extensively across geographical areas due to the distinct lifestyles and exposure to environmental carcinogens [49]. These reports seem to support our findings of slightly lower risk of cancer associated with rs2294008 genotypes, compared to that reported in a previous association study of H. pylori infection [36]. This difference indicates that rs2294008 polymorphism relative to other carcinogens, such as infection with H. pylori, contributes a less part to carcinogenesis. The aetiology of cancer is multifactorial and heterogeneous, further investigation is needed to identify the risk factors, thus facilitating an early detection and prevention.

There is significant between-study heterogeneity in this meta-analysis. Although no substantial change was found in the combined results when removing the study donating the heterogeneity, we can not rule out the potential influence and the current findings should be treated with caution. Furthermore, in terms of the moderate sample size in stratified analyses, it is still indecisive about the stable effect size of *PSCA* rs2294008 polymorphism in connection with cancer risk. Finally, departure from HWE was detected in two studies. By comparing the results between including and excluding them, we observed minor alternation in the results failing to reach the significant level. Nevertheless, in light of few significant effects as a result of the presence of heterogeneity and departure form HWE, our findings are reliable based on the statistical evidence.

In summary, this meta-analysis suggested that *PSCA* rs2294008 polymorphism was significantly associated with increased risk of cancer. Stratified analyses in cancer type and ethnicity showed similar results. However, it is worthwhile to carry out large studies in future to validate the present findings.

## Supporting Information

S1 PRISMA Checklist (DOC)
